# Supplementary material for: Matching sensor ontologies through siamese neural networks without using reference alignment
Source: PeerJ Comput Sci. 2021 Jun 18;7:e602. doi: 10.7717/peerj-cs.602 (PMC8237319; doi:10.7717/peerj-cs.602)
Supplement: Supplemental Information 1 [file peerj-cs-07-602-s001.zip › 262/onto.html]

Author: Nick Knouf <nknouf@mit.edu>  
Contributor: Antoine Zimmermann <antoine.zimmermann@inrialpes.fr>, Jérôme Euzenat,   
Date: 08/06/2005  
Version: $Id: onto.rdf,v 1.30 2008/05/27 14:41:13 euzenat Exp $

## Classes

**http://www.w3.org/1999/02/22-rdf-syntax-ns#List** (, *)*


**http://xmlns.com/foaf/0.1/Person** (, *)*


**http://xmlns.com/foaf/0.1/Organization** (, *)*


**sqdsq** (, *)*


**hazdn** (, *)*


**zajdhqs** (, *)*


**dzajndsq** (, *)*


**sqxsqkd** (, *)*


**qsdsnbsqd** (, *)*


**zdzaudbd** (, *)*


**sqdsopq** (, *)*


**dzqndbzq** (, *)*


**vccfsq** (, *)*


**dcsqdcsqd** (, *)*


**deqdxcsqcsq** (, *)*


**scds** (, *)*


**dcsqdsq** (, *)*


**zdazsx** (, *)*


**xsqlknk** (, *)*


**zqdzqzai** (, *)*


**wxsdxqsdh** (, *)*


**zdqssqdb** (, *)*


**dqzdxdcsqj** (, *)*


**sdcsqhyz** (, *)*


**azzauhuie** (, *)*


**zeadza** (, *)*


**dscdscg** (, *)*


**qsdsquj** (, *)*


**zqedzbx** (, *)*


**qzddj** (, *)*


**izxnquo** (, *)*


**zauio** (, *)*


**zadazxn** (, *)*


**dsqdbz** (, *)*


**dnqsb** (, *)*


**dzshyuua** (, *)*

## Properties

**http://www.w3.org/1999/02/22-rdf-syntax-ns#first**: http://www.w3.org/1999/02/22-rdf-syntax-ns#List -> \_ *()*


**http://www.w3.org/1999/02/22-rdf-syntax-ns#rest**: http://www.w3.org/1999/02/22-rdf-syntax-ns#List -> http://www.w3.org/1999/02/22-rdf-syntax-ns#List *()*

**http://purl.org/dc/elements/1.1/creator**\_ -> \_ *()*


**http://purl.org/dc/elements/1.1/contributor**\_ -> \_ *()*


**http://purl.org/dc/elements/1.1/description**\_ -> \_ *()*


**http://purl.org/dc/elements/1.1/date**\_ -> \_ *()*


**http://xmlns.com/foaf/0.1/firstName**\_ -> \_ *()*


**http://xmlns.com/foaf/0.1/name**\_ -> \_ *()*

## Individuals

<rdf:List@ttp://www.w3.org/1999/02/22-rdf-syntax-ns#nil>

---

Generated by OWL2HTML
